# Supplementary material for: Are carbon emissions trading and green financial instruments synergistic? -Comprehensive quantitative research based on content analysis
Source: PLoS One. 2024 Mar 7;19(3):e0298601. doi: 10.1371/journal.pone.0298601 (PMC10919601; doi:10.1371/journal.pone.0298601)
Supplement: S1 Data — (ZIP) [file pone.0298601.s001.zip › Data/地级市Z_F工作报告.zip!/╔┬╬≈/═¡┤¿╩╨/└┤╫╘╣1⁄2╓┌║┼í╛┬φ┐╦╩2╛▌═°í┐_2017.docx]

各位代表：

　　现在，我代表市人民政府向大会报告工作，请予审议，并请市政协委员和其他列席人员提出意见。

　　一、2016年和本届政府工作回顾

　　2016年是“十三五”开局之年，也是本届政府任期的最后一年。面对错综复杂的宏观经济环境和前所未有的下行压力，在市委的坚强领导下，全市上下认真贯彻落实中省决策部署，深入践行新发展理念和“五个扎实”要求，紧扣追赶超越、转型发展，突出抓好“两稳两优两增”，凝心聚力、迎难而上，全市经济发展保持缓中趋稳、稳中向好，较好地完成了市十五届人大八次会议确定的目标任务。实现生产总值311.61亿元、增长7%，地方财政收入21.51亿元、同口径增长10.04%，城乡居民人均可支配收入27594元、9478元，分别增长8%、8.5%。

　　（一）坚持不懈抓项目，投资平稳较快增长。及时出台重大前期项目工作管理办法、加强招商引资的意见和扩大有效投资考核奖惩办法，组织了项目集中开工、项目观摩和“百天百项百企百亿”投资促进工作。全年招商引资签约项目162个，到位资金177.97亿元。开工项目102个。100个市级重点项目完成投资165亿元，占年度任务的103%。包海线西安—延安—榆林高铁西延段项目开钻，华能铁路专用线建设基础工作启动，美鑫锦阳电厂1、2号机组等26个项目建成投运。全社会固定资产投资完成423.23亿元，增长10.2%。

　　（二）精准施策稳工业，工业经济缓中有进。面对多重困难叠加的严峻挑战，先后出台工业提质增效10条措施和稳增长促投资15条意见，努力消减煤矿停产整顿、“去产能”对工业经济的冲击。铜铝公司全面复产，铝业增长2.1倍，电力、水泥分别增长31.8%、11.3%。装备制造、食品加工、医药生产等接续产业增加值增长36.1%。4个市级、4个县域工业园区建设步伐加快，董家河循环经济产业园集群项目进展顺利，坡头工业园区列入全国增量配电业务改革试点。新增规模以上工业企业38户。全市规模以上工业增加值完成133.93亿元，增长6%。

　　（三）围绕增收优农业，美丽乡村亮点增多。政策性小麦、玉米保险全覆盖，粮食总产24.17万吨，实现“十三连丰”。建成市级水果示范园19个，水果总产75.57万吨、增长3.5%。“铜川苹果”荣获第十四届中国国际农产品交易会金奖，“铜川大樱桃”被指定为2016年G20农业部长会议专用果，印台区被评为全国现代苹果产业30强县。15个农业科技项目获得杨凌农高会后稷特别奖，步寿塬现代农业园区创建为省级科技示范园区，席地园农民专业合作社评为全国示范社，7家农民专业合作社评为省级百强示范社。编制了“十三五”美丽乡村建设专项规划，完成5个美丽乡村示范片区和60个创建村建设任务，实施项目469个，完成投资9.9亿元。

　　（四）突出特色强旅游，第三产业较快发展。60个旅游重点项目完成投资16亿元。孙思邈纪念馆建成开放，照金红色旅游名镇、玉华宫景区旅游服务设施入选全国优选旅游项目名录，宜君旱作梯田被授予省级农业生态博物馆。全年接待游客1395万人次，旅游总收入76.4亿元，分别增长15%和16.5%。全面启动了区县、乡镇、村级电子商务服务机构建设，耀州区荣获全国电子商务进农村示范区。全社会消费品零售总额、服务业增加值分别增长13.6%和8.5%。

　　（五）弥补短板拓功能，城乡统筹步伐加快。启动了“多规合一”试点，修编了风景名胜区总体规划，建成城市地下管线信息动态管理系统，开展了省级海绵城市建设试点。210国道改扩建、华原西道等工程进入扫尾阶段，新区东环线建成通车，西北环线开工建设。南市区集中供热工程如期投运，供热面积395万平方米。照金镇入选中国特色小镇，红土镇、哭泉镇列入省上跟踪指导考核重点镇。

　　（六）硬法重拳治污染，生态环境逐步好转。治污降霾网格化监管体系基本建成。拆除了33家关闭污染企业的废弃建筑物，开展了以治理臭氧污染为主的“百日行动”，淘汰黄标车1469辆。全年空气质量优良天数210天，排关中五市第二。实施漆水河、沮河城市段综合治理，完成10个乡镇24个村的环境综合整治示范项目。全年植树造林11.08万亩。万元生产总值能耗下降3.92%，四项主要污染物减排指标超额完成。省级环保模范城市创建通过技术评估，王益区、印台区分别获得全国、全省绿化模范区。

　　（七）蹄疾步稳促改革，发展动能不断提升。完成年度改革任务110项。制定了供给侧结构性改革工作措施和专项行动计划，引导退出煤矿21处，淘汰落后产能1058万吨，分别占全省的34%、36%。房地产“去库存”51.02万平方米。制定了加强财源建设的意见和激励考评办法，设立了27亿元财经发展基金，8个项目入选国家PPP示范项目，占全省的三分之一。西安银行和新华、恒通村镇银行落户铜川，宜君县被列为全国唯一的县级农村普惠金融综合示范区。中省取消下放行政审批事项承接落实到位，市级行政审批事项精简率44.7%，新增市场主体6032户。全面推进“营改增”试点，累计减税8039万元。不断深化与韩国奉化郡、美国约巴琳达市的友城关系，举行了“一带一路”建材产业对接会，全市外贸进出口总额2370万美元。

　　（八）精准务实抓脱贫，群众生活得到改善。民生支出占财政总支出的80.05%，较上年增加4.64亿元。按照“六个精准”，扎实推进“五个一批”脱贫攻坚，开展了“冲在一线、干在实处、帮贫解困促发展”行动，移民（脱贫）搬迁4186户，2.8万贫困人口脱贫。城镇新增就业10103人，登记失业率3.3%。提高了机关、企业退休人员基本养老金水平，城乡居民医保财政补助标准年人均提高40元，社保卡持卡人数48.04万人。十三年免费教育全面实施，472套农村学校教师周转房开工建设。我市被确定为国家医养结合试点单位，北京中医药大学第七临床医学院暨孙思邈医院落户铜川。成功举办了市第三届文化艺术节。食品药品安全监管不断加强，宜君县创建为省级食品安全示范县。安全生产隐患大排查大整治不断深入，社会治安进一步加强，信访工作综合排名全省第一。

　　（九）完善机制转职能，机关作风明显转变。巩固拓展党的群众路线教育实践活动和“三严三实”专题教育成果，深入开展“两学一做”学习教育，认真落实“三项机制”，促进政府职能和机关作风“双转变”。我市被评为全国法治宣传教育先进市。办理人大代表建议82件、政协提案184件。全面公开区县、市级部门预决算，全市“三公经费”较上年下降22%。清理不符合现行法律法规、不适应发展的文件322份。2397名公民代表走进政府，60名公众代表列席政府会议，300名大学生到机关见习。办结人民网留言200条、“市长信箱”来信2053件，“铜川发布”获得陕西政务微信创新突破奖。

　　各位代表，2016年全市经济社会发展目标任务的完成，标志着本届政府的圆满收官。这六年，是我市发展进程中不平凡的六年，经历了能源、建材市场的剧烈波动，经历了发展方式、体制机制的深刻变革，经历了速度换档、结构调整、动力转换的重大变化，经历了罕见的干旱和强暴雨等自然灾害，全市上下众志成城、攻坚克难，战胜了各种困难和挑战，顶住了经济下行压力，较好地完成了“十二五”目标任务，实现了“十三五”良好开局，“四个铜川”建设迈出坚实步伐！

　　这六年，是我市换档调速、争先进位、综合实力显著提升的六年。生产总值是2010年的1.7倍，年均增长12%，高于全省1.4个百分点；固定资产投资累计完成1743亿元，年均增长24%；地方财政收入是2010年的1.6倍，年均增长8.1%。在全省县域经济社会发展综合考评中，宜君县较2010年进了20位，王益区进了4位，耀州区进了3位，都先后获得全省争先进位奖。我市在2015年度全省目标责任考核中获得优秀格次。

　　这六年，是我市结构调整、产业优化、转型发展明显加快的六年。8个工业园区被确定为省级重点县域工业集中区，董家河循环经济体系初步形成。装备制造、食品加工、医药生产等接续产业加快发展，非煤产业占比88%。游客接待量、旅游总收入年均分别增长20%、32%。服务业占比提高11.3个百分点。在全国资源枯竭型城市转型、节能减排财政政策综合示范城市绩效考核中，均获得优秀格次。

　　这六年，是我市城乡统筹、环境优化、城乡面貌变化较大的六年。城镇化率由2010年的57.9%提高到64.2%。西铜、铜旬高速和耀旬二级公路、210国道改扩建等工程建成通车，拉大了城市骨架。照金、董家河、黄堡、陈炉、彭镇被确定为全国重点镇。成功创建为全国绿化模范城市、国家卫生城市，被命名为中国循环经济十佳绿色发展城市。

　　这六年，是我市深化改革、动能转换、发展活力不断增强的六年。“放管服”改革深入推进，清理取消124项、下放28项行政审批事项，有效激发了市场活力。非公经济占比提高7.8个百分点。国有企业改革有序推进，完成秦岭水泥公司、市天然气公司等企业改制重组。建成四级便民服务体系，打通了服务群众“最后一公里”。

　　这六年，是我市民生改善、社会和谐、群众福祉持续增进的六年。城乡居民人均可支配收入年均分别增长12.3%、14.7%，农村居民收入增速连续七年快于城镇居民。累计新增就业8.4万人，城镇登记失业率控制在4%以内。建成保障性住房7.1万套，城镇居民人均住房面积由2010年的30.29平方米增加到42.8平方米。城乡居民养老和医疗保险参保率稳定在98%以上，群众的获得感、幸福感显著增强。

　　各位代表，过去六年的成绩来之不易，这是省委、省政府和市委正确领导的结果，是全市各条战线共同努力的结果，凝结着历届四大班子的智慧和心血。我代表市人民政府，向全市广大干部群众，向驻铜部队指战员、武警官兵、政法干警和中省在铜单位，向大力支持政府工作的各位代表、各位委员和社会各界人士，表示崇高的敬意和衷心的感谢！

　　回顾总结这六年来的奋斗历程，我们深感有以下体会：

　　第一，必须系统推动转型发展。发展是第一要务，转型发展是新发展理念在铜川的具体实践。只有围绕循环链部署产业链、创新链和资本链，系统推进产业、城市、社会、体制机制转型，才能实现更高质量、更有效率、更加公平、更可持续的发展。

　　第二，必须坚持项目带动战略。抓项目就是抓发展，发展靠项目、项目靠实干。我们必须把项目建设作为经济工作的主要抓手，做到任务项目化、项目数字化、抓项目常态化。只有精心策划项目、全力招引项目、聚力推进项目，追赶超越、转型发展才有硬支撑。

　　第三，必须加大改革创新力度。惟改革者进，惟改革创新者胜。我们必须把改革创新贯穿经济工作的全过程，用足用好中省各项政策，最大限度释放体制机制红利。“改革”是名词更是动词，只有坚持问题导向，纵深推进改革开放，破除机制藩篱，才能充分迸发发展活力。

　　第四，必须树牢共享发展理念。让群众过上好日子，是政府一切工作的出发点和落脚点。我们必须坚持“两个80%”不动摇，抓住群众需求的“最大公约数”，尽力办好民生实事，做到一切为了人民、发展惠及人民。只有牢固树立以人民为中心的发展思想，各项事业才能获得不竭力量。

　　第五，必须聚合力量推动发展。实践充分证明，只有全市一盘棋、上下同担当、条块相结合、同向共发力，聚焦经济主战场，低调务实不张扬，层层传导压力、级级压实责任、人人使劲给力，才能汇聚起追赶超越的强大合力。

　　这些经验来自于发展实践，弥足珍贵，我们应当倍加珍惜并长期坚持。在看到成绩的同时，我们也清醒地认识到，我市发展中还存在不少困难和问题。主要是：受经济大环境和自身结构影响，我市传统产业抵御市场风险能力较弱，稳增长的压力持续加大；创新驱动能力不够强，第三产业发展滞后，接续产业规模小，民间投资活力不足，经济外向度较低，新经济、新业态发展还不理想；城市服务功能还不完善，资源环境约束趋紧，城乡统筹发展、环境保护和生态建设的任务更加繁重；脱贫攻坚任务艰巨，文化教育、医疗健康、社会保障、劳动就业等领域仍有群众不满意的地方；面对新形势、新任务，一些政府工作人员的责任担当、创新意识、能力水平还有差距。我们必须以事不避难的勇气和智慧，直面问题、克服困难、破解难题，决不辜负全市人民的信任和期盼。

　　二、未来五年追赶超越的总体思路和目标

　　习近平总书记提出“陕西正处在追赶超越阶段”的重要论断，是放眼全球大势和国家大局，对陕西发展态势和所处历史方位作出的科学判断。全省牢记重托、奋力追赶、竞相超越的局面已经形成。我市作为关中城市群成员，前有标兵、后有追兵，必须把追赶超越、转型发展作为当前和今后一个时期全市的中心任务，抓住难得机遇，锐意改革创新，拓展发展思路，实现在全省市区中争先进位、在全国资源转型城市中追赶超越。

　　目前，我市正处于追赶超越、转型发展的关键节点，机遇与挑战并存，但机遇大于挑战。国家实施更加积极有效的财政政策，保持稳健中性的货币政策，深入实施西部大开发和“一带一路”战略，批准设立陕西自贸区，为我市加强基础设施建设、优化产业结构、扩大对外开放带来了十分难得的机遇。铜川作为资源转型和节能减排国家级试点城市，又入选全国山水林田湖生态保护修复项目示范区，西安至延安高铁、合阳至铜川高速等重大项目将陆续开工建设，关中协同创新发展加快推进，为我市积蓄了强大的发展势能。“两学一做”学习教育深入开展，干部群众人心思进、人心思干，追赶超越、转型发展其势已成。

　　我市“十三五”规划描绘了全面建成小康社会的蓝图，市十二次党代会对追赶超越、转型发展进行了全面部署。未来五年，全市经济社会发展的总体思路是：高举中国特色社会主义伟大旗帜，坚持以马克思列宁主义、毛泽东思想、邓小平理论、“三个代表”重要思想、科学发展观为指导，全面贯彻党的十八大和十八届三中、四中、五中、六中全会精神，深入学习贯彻习近平总书记系列重要讲话精神，全面落实省委、省政府各项决策部署和市十二次党代会精神，紧扣“五位一体”总体布局、“四个全面”战略布局和“五个扎实”的要求，坚持稳中求进工作总基调，以新发展理念为引领，以追赶超越、转型发展为主线，以推进供给侧结构性改革为抓手，以全面从严治党为保障，以在全省早日全面建成小康社会为目标，走对路扎实干，努力开创铜川各项事业新局面。

　　总体发展目标：一是经济保持稳步增长。生产总值年均增速高于全省1个百分点以上，科技研发经费、战略性新兴产业增加值、文化产业增加值、居民收入追赶全省平均水平，社会消费品零售总额、规模以上工业增加值、地方财政收入、服务业增加值增速超越全省平均水平。二是产业结构不断优化。非煤产业占比达到92%，服务业占比达到42%。三是城乡协调发展。城市功能不断完善，城镇化率达到71%。生态文明建设取得显著成效，创建国家园林城市、国家森林城市、全国文明城市。四是民生改善大提升。居民人均可支配收入年均增长9%以上，美丽乡村、新型农村社区建设稳步推进，农村生产生活条件切实改善，2018年贫困区县如期摘帽，贫困村、贫困人口全部脱贫。社会保障不断完善，群众享有更便利、更普惠、更优质的公共服务资源。

　　实现这些目标，必须牢牢把握“五个扎实”的赶超路径。扎实推动经济持续健康发展，坚持内涵提升与外延发展并重，按照传统产业循环化、接续产业高端化、产业布局集约化，积极实施创新驱动，发展壮大实体经济，加强区域协同创新，全力扩总量、提质量、增效益。扎实推进现代农业建设，坚持区域化布局、产业化带动、园区化示范、规模化发展，突出发展特色农业，加快发展农业园区，打响铜川农业品牌。扎实加强文化建设，深度挖掘历史文化资源，唱响药王养生文化，突出文旅融合发展，做优做强文化旅游健康服务产业，创建国家公共文化服务体系示范区，打造全国全域旅游示范市。扎实做好保障和改善民生工作，面向大多数、紧盯最底层，持续用力改善民生，创建教育强市、健康城市，争创国家创新创业城市，全面推进生态文明建设，努力营造安定、有序、和谐的社会环境，不断提高人民群众幸福指数。扎实落实全面从严治党，围绕中心、服务大局，全面加强政府系统党的建设，抓好人才培养和引进，全面提升干部素质和能力，为追赶超越提供强有力的保障。

　　各位代表，追赶超越、转型发展是沉甸甸的历史使命，我们一定要坚定发展自信，咬定目标不放松、撸起袖子加油干，努力闯出转型升级的加速度和致胜未来的新优势，奋力谱写追赶超越、兴铜富民新篇章！

　　三、2017年的主要工作任务

　　今年是党的十九大和省第十三次党代会召开之年，是实施“十三五”规划、奋力追赶超越、决胜全面小康的关键一年，也是推进供给侧结构性改革的深化之年。做好今年经济社会发展工作至关重要，意义重大。我们认真贯彻落实市委十二届二次全会精神，牢固树立和践行新发展理念，坚持稳中求进工作总基调，按照“五个扎实”要求，以提高发展质量和效益为中心，以推进供给侧结构性改革为抓手，突出“四轮”驱动，强化“三区”支撑，扩总量、提质量、增效益，调结构、惠民生、补短板，促进经济平稳健康发展、社会和谐稳定。

　　综合考虑各方面因素，今年经济社会发展的主要预期目标是：生产总值增长9%左右，规模以上工业增加值增长8%左右，地方财政收入增长7%左右，全社会固定资产投资增长15%左右，服务业增加值增长10%左右，全社会消费品零售总额增长13.5%左右，城乡居民人均收入分别增长9%和10%左右，城镇登记失业率控制在3.9%以内，CPI涨幅控制在2.5%左右，万元生产总值能耗、主要污染物减排完成省上下达的指标。

　　（一）坚持项目带动战略，筑牢追赶超越根基

　　强力推进重点项目。今年实施在建市级重点项目120个，总投资862.17亿元，年内完成投资215亿元；策划包装单体投资额10亿元以上的项目不少于10个，新增储备入库项目不少于100个，策划项目总投资不少于1000亿元。确保川陈旅游公路、宜君油气资源开发等80个续建项目达到序时进度，美鑫锦阳电厂3、4号机组、龙潭水库大坝等30个项目建成投运，积极推进合阳至铜川高速等40个项目尽快开工建设。全力保障西延高铁铜川境内各项工作，抓好华能照金电厂铁路专用线建设。认真落实服务重点项目建设相关要求，继续采取市级领导包抓，区县、部门、驻工地专员和业主合力推进的工作机制，组织好项目开工和项目观摩活动，观摩对象由区县、工业园区扩大到景区。

　　集聚要素保障项目。制定稳定金融支持、激活民间资本的措施，充分释放金融和社会资本潜力。加强政银企合作，发挥财经发展基金的撬动作用，吸引更多社会资本参与项目建设，加快推进PPP示范项目落地实施。完善项目供地制度，提高用地审批效率，加快闲置、低效土地清理，依法收回违规用地，确保稀缺资源优先保障重点项目建设。利用好项目在线审批平台，实行并联审批，缩短项目生成周期。夯实项目建设的责任，兑现激励奖补政策，严格问责问效，形成抓项目建设的合力。

　　择优选强招引项目。加大精准招商力度，加强与知名企业、行业协会、商会的联系对接，采取专业招商、定向招商、以商招商等方式，全力招引能够形成全产业链的旗舰型企业，带动关联产业发展。组织开展专题招商，积极参加省内外重大招商活动，办好第四届中国孙思邈中医药文化节，力促一批大项目、好项目落地。健全投资环境评价机制和社会监督体系，坚持重点投资企业定期回访制度，营造亲商、安商、富商的投资环境。全年实际利用市域外资金180亿元以上。

　　（二）多措并举稳定增长，促进工业提质增效

　　强化工业运行协调。认真落实稳增长各项政策措施，及时准确研判市场变化，对煤、电、铝、水泥等主导产业和50户重点工业企业，加强调度、分类施策、全程服务，在确保质量和效益的前提下，促进主导产业、骨干企业稳定增长。采取大矿托管、减量置换等办法，提升煤炭安全供给能力。对成长性好的中小企业、小微企业，加大政策、信贷担保等扶持力度。全年新增规模以上工业企业20户。

　　加快工业转型升级。大力实施创新驱动战略，围绕“中国制造2025”及陕西实施意见、关中协同创新发展，加快市科技资源统筹中心建设，发挥企业创新主体作用。按照“精煤、强铝、扩电、优水泥”思路，加快传统产业改造提升。围绕打造关中新型能源基地，积极实施精洗煤等洁净能源项目，有序发展光伏、风电等项目，构建煤电、光伏、风能多元电力体系。抓住陕西航空航天和百万辆汽车工程，加强与中航、西飞、秦川、陕汽、比亚迪、吉利等企业对接，主动提供协作配套，培育发展工业铝型材、汽车铸配件，努力打造陕西航汽铝材料工业和汽车零部件生产基地。做强做优水泥产品，加快推进陕建工集团新型材料工业园建设，积极开发工程预制件等水泥制品；发挥耀瓷品牌优势，大力发展日用陶瓷、艺术陶瓷、建筑陶瓷和卫生陶瓷，打造陕西新型建材基地。加快培育发展装备制造、食品加工、医药生产等业态，提高接续产业对经济增长的贡献份额。积极实施品牌战略，发扬工匠精神，大力推进名牌培育、名牌保护，不断提升供给体系质量和效率。

　　壮大工业园区实力。明晰园区产业定位，发挥比较优势，加快建设承载工业、引领转型的产业集群发展新高地。配套服务向园区集成，支持政策向园区倾斜，生产要素向园区聚集，尽快弥补基础设施保障不足、产业性项目缺乏的短板。创新新区南部工业园区发展机制，大力发展高新技术产业，加快培育现代制造、高端铝材等产业，把铜川新区创建为省级高新技术开发区。围绕深度融合、协同配套，加快董家河循环产业集群项目建设，尽快闭合循环产业链。抓好黄堡工业园区老企业闲置资产盘活，加快推进陶瓷文化园区建设。推进坡头载能工业、建材、物流园等项目建设，提升园区整体发展水平。加快推进用电大户直供电政策落实，努力把坡头、董家河园区打造成低电价区。抓好县域工业园区建设，加快培育园区竞争新优势。

　　（三）加快现代农业建设，做优做强特色农业

　　大力发展现代农业。认真贯彻落实中央一号文件精神，以农业增效、农民增收、农村增绿为重点，大力推进农业供给侧结构性改革，促进农村一二三产业融合发展。严守耕地红线，新建基本农田1.5万亩，发展高效节水灌溉农田1万亩。加强农业防灾减灾体系建设，提高农业综合生产能力，粮食总产稳定在20万吨以上。依托农业产业化龙头企业带动，持续抓好5个现代农业综合示范区建设，加快推进12个省级、9个市级、20个区（县）级现代农业园区建设。大力实施果业品牌战略，推进适度规模的标准化生产经营，提高果业综合效益。抓好规模养殖场改造，发展高效养殖和生态循环农业。开展农业废弃物资源循环利用试点。学习借鉴河南许昌经验，结合美丽乡村建设和园林城市、森林城市创建，按照市场化运作的思路，积极发展苗木花卉产业，带动休闲观光农业发展。大力推进“互联网＋”现代农业行动，支持农产品电商平台和乡村电商服务站点建设，实现农村电子商务全覆盖。全年培育农业产业化龙头企业10户以上，发展示范家庭农场20家。

　　加快建设美丽乡村。深化农村综合改革，开展农村集体产权制度改革试点，落实农村土地集体所有权、农户承包权、土地经营权“三权分置”政策。统筹推进农村土地征收、集体经营性建设用地入市、宅基地制度改革，鼓励引导土地承包权有偿退出、土地经营权入股农业产业化经营，做好承包土地经营权和农民住房财产权抵押贷款试点。巩固提升美丽乡村建成片区和建成村建设水平，新建5个示范片区和80个美丽乡村。加快实施农村公路安全生命防护工程，改善农村公路230公里。解决2.5万农村居民饮水安全问题。深入开展农村环境整洁行动，重点实施生活垃圾、污水治理和改厕、取暖工程，改善农村生态、人居和发展环境。

　　倾力推进脱贫攻坚。不断完善“1＋3＋N”政策体系，确保各项精准脱贫政策落地生根。统筹整合使用财政涉农资金，各类涉农资金和惠民政策向贫困地区倾斜，加快推进照金片区、铜罕沿线、金锁西片、洛河西岸四个重点区域脱贫攻坚。把产业扶贫作为重中之重，精准产业扶贫措施，夯实脱贫致富基础。做好科技扶贫、金融扶贫、旅游扶贫，分类推进教育扶贫、健康扶贫，移民（脱贫）搬迁工程基本建成。积极开展保险助推脱贫省级试点工作，筑起因病因灾致贫返贫的安全防线。年内实现85个贫困村、1.4万贫困人口脱贫，宜君县脱贫摘帽。

　　（四）抓好文化旅游产业，加快推动服务业升级

　　加快发展旅游业。挖掘文化资源，讲好铜川故事，推进全域旅游，建设全景铜川。加强与陕文投集团、陕旅集团、龙之旅集团等知名企业合作，精心打造照金、玉华宫、药王山、陈炉四大景区，启动照金5A级景区创建。扎实做好耀州国家全域旅游示范区、宜君省级旅游示范县建设，巩固提升马咀休闲、金锁石林、哭泉梯田等乡村旅游点，抓好郭秀明教育基地和新区慢谷、孟姜女故里秦人村落、战国魏长城等文化景点建设，加快王石凹工业遗址文化景区建设。办好陕甘边红色旅游联盟铜川年会。全年完成旅游投资12亿元以上，游客接待量、旅游总收入分别增长12%和15%。

　　提升商贸服务业。推动商业形态创新，加快推进商业综合体建设，荣盛国际街区、美佳华商业中心投入运营。采取“房东变股东”、为创业者提供孵化器等方式，积极盘活存量商业用房。加快陕西现代果业物流园、西北耐物流园建设，打造区域性商品交易中心。大力实施电子商务示范工程，积极创建国家和省级电子商务示范园区和企业，加强与知名电商合作，促进包装、配送、快递等服务业发展。

　　培育消费新热点。发挥国家级医养结合试点的优势，全面启动健康城市建设工作。加强与北京中医药大学、中国中医药科技开发交流中心、北京同仁堂等合作，推进中医药创新创业公共服务平台建设，加大医药产品研发，扶持医药企业发展。加快发展旅游、文化、体育、健康、养老五大幸福产业，促进消费提质扩容。鼓励发展便民利民社区商业，打造“15分钟”生活服务圈，努力释放消费潜力。

　　（五）推动新型城镇化建设，全面提升城市品质

　　强化城市规划管控。顺应现代城市发展新理念，加快推进“多规合一”，提升城市空间规划和建筑设计水平。做好城市总体规划编制，启动向乡镇、农村延伸的城乡一体化规划和管理。强化规划的战略引领和刚性约束，扎实开展违法建设专项治理，推动城镇化健康发展。

　　完善城市承载功能。强化经营城市理念，加快推进绿色智慧人文城市建设。按照“先地下、后地上”的要求，加强道路交通设施建设，加快打通“断头路”，稳步推进海绵城市和地下综合管廊建设，做好城市防内涝工作。推进非机动车道、城市步道、无障碍设施建设，改造人行道2万平方米。加强集贸市场、公园绿地、停车场等规划建设。加快推进供水、供气老旧管网改造，提高城市集中供热管网覆盖率，新增天然气用户8000户。实施新型智慧城市建设，建成综合服务“一号通”、无线城市“一网通”、大数据综合市场监管等服务系统。扎实推进国家园林城市、省级生态园林城市创建，策划包装新区水系建设项目，新增城市绿地30万平方米。

　　提高城市管理水准。推进城市管理执法体制改革，组建城市管理综合执法部门，理顺市、区两级城市管理职责。健全网格化管理模式，加强徒步街巷现场管理及巡查考核工作，进一步提升城市管理的精细化水平。完善各项保障机制，促进污水处理厂和垃圾处理场稳定运行。巩固提升创国卫成果，确保顺利通过国家复审。启动全国文明城市创建工作，提升城市文明程度和市民文明素质。加强住宅小区物业管理，提升城市社区治理服务水平。

　　加快特色城镇建设。放大耀州城乡统筹发展试点效应，加快推进城乡协调发展。支持宜君秀美山城建设。突出抓好黄堡重点示范镇和陈炉、照金文化旅游名镇及董家河、红土、哭泉省上跟踪指导考核重点镇建设，有序推进20个建制镇建设，不断增强城镇综合承载能力，促进农业转移人口就地就近市民化。

　　（六）加强生态文明建设，共建共享绿色家园

　　加大环境保护力度。严格执行《环境保护法》《大气污染防治法》和铜川环保“铁十条”，打好大气、水体、土壤污染治理三大战役。把治霾作为环保头号工程，深入开展“铁腕治霾·保卫蓝天”行动，加大减煤、控车、抑尘、禁燃、增绿等工作力度，抓好源头防控，促进空气质量稳步提升。认真落实渭河流域水污染防治行动方案，确保出市断面水质达标。开展土壤环境质量类别划定工作，推进土壤污染防治。加强自然资源环境审计、环保督察巡查工作，强化“党政同责、一岗双责”，强化环保部门监管执法责任，强化企业清洁生产责任，严肃追究失职渎职和环保违法行为。

　　持续用力修复生态。系统推进山水林田湖生态保护修复工程建设，筑牢生态安全屏障。启动国家森林城市创建，持续抓好城市、园区、景区、道路、河流、庭院等重点区域造林绿化，全年植树造林12万亩。实施好3个矿山地质环境恢复治理项目，启动川口至青岗岭区域生态修复治理工程。全面实行河长制，加强水资源保护、水域岸线管理、水污染防治和水环境治理。完成漆水河、沮河综合治理任务，启动赵氏河流域综合治理，加快赵氏河、福地湖国家湿地公园建设。治理水土流失150平方公里。

　　节约集约利用资源。实施能源和水资源消耗、建设用地等总量和强度双控行动，推进绿色低碳循环发展。巩固节能减排财政政策综合示范市创建成果，切实抓好工业、交通、建筑和公共机构节能。强化水资源“三条红线”意识，提高中水回用率。统筹利用城乡建设用地指标，做好工矿废弃地复垦利用工作。

　　各位代表，良好的生态环境是最普惠的民生福祉。我们要牢固树立“绿水青山就是金山银山”的意识，以对人民群众、对子孙后代高度负责的态度和责任，呵护好绿色家园，让群众享受到更优的生态福利！

　　（七）扎实推进改革开放，发挥改革牵引作用

　　深化供给侧结构性改革。从严控制产能过剩行业新增产能项目，坚决淘汰不达标煤矿。制定处置“僵尸企业”工作方案，落实好相关政策措施，有序引导退出市场。建立健全购房与租房相结合、市场配置与政府保障相补充的住房制度，积极推进棚户区改造货币化安置，释放农业转移人口购房需求，有效化解商品房库存。推进市场化债权股权转换，加大股权融资力度，降低企业负债率和不良贷款率。深入开展企业降本增效行动，从税费、融资、制度性交易、用能用地、内部挖潜等方面减负，促进企业轻装上阵增效益。从解决制约经济社会发展的薄弱环节和突出问题入手，着力补齐发展短板，不断增强发展的协调性。

　　抓好国企国资等领域改革。制定市属国有企业整合方案，探索开展国有资本投资运营试点，积极发展混合所有制经济，支持国有、集体、非公资本交叉持股、相互融合。启动国有企业“三供一业”移交工作，加快剥离企业办社会职能。推进产权制度改革，加强对各种所有制组织和自然人财产权的保护。深化财税体制改革，加强财源建设，提高财政收入质量，完善预算管理制度，增强财政保障能力。利用好中国人民银行普惠金融试点的机遇，加快完善金融服务体系，创新金融产品和服务。做好投融资体制、盐业体制、国有林场等改革。

　　深入推进“放管服”改革。完善权力、责任清单管理和动态调整工作，强化事中事后监管，以更大的力度为市场松绑、为企业减负、为实体经济服务。构建“亲”“清”新型政商关系，培育、保护企业家精神。搞活国有企业，扶持民营企业，培育小微企业、科技型企业、瞪羚企业，积极发展总部经济，努力振兴实体经济。鼓励、支持和引导非公经济发展，非公经济占比达到51.8%。

　　积极扩大对外开放。加强与“一带一路”沿线地区的交流合作，主动融入陕西自贸区建设大格局，更好统筹“引进来”与“走出去”，不断提升铜川经济的外向度。借助西安国际港务区平台，大力培育外贸骨干企业，打造外贸特色品牌，扩大建材、陶瓷、苹果、中药材等地产品出口。全年外贸进出口总额2600万美元。

　　（八）全力保障改善民生，促进社会和谐稳定

　　保持就业稳健增长。完善“双创”服务体系，提升就业服务能力，全面完成省级创业型城市创建工作。新建一批专业化众创空间，促进大众创业、万众创新，全年发放创业担保贷款2亿元。做好高校毕业生、农村转移劳动力、城镇困难人员、退役军人和残疾人创业就业，稳妥做好“去产能”企业职工分流安置工作。城镇新增就业9000人以上。

　　健全社会保障体系。完善被征地农民、个体从业人员、农民工等群体参加社会保险政策。实行统一的城乡居民基本医疗保险制度，启动实施异地就医住院医疗费用直接结算，完善重大疾病救助办法，实现职工医保、居民医保、大病保险、医疗救助“一站式”办理。建成5个城市社区服务体系项目、25个农村社区服务中心。完成市老年服务中心主体工程建设，新建72个农村幸福院、5个社区日间照料中心。做好农村五保户、城市“三无”人员、高龄和失能老人、残疾人、计划生育特殊家庭的生活保障，关爱农村留守儿童、妇女和老人，支持慈善事业发展。

　　提升教育医疗服务水平。全面实施教育“六大工程”，促进各类教育协调发展。启动实施第三期学前教育行动计划，推进义务教育均衡发展，提升普通高中教育质量。印台区义务教育均衡发展和“双高双普”创建工作通过评估验收，我市在全省率先实现全覆盖。资助400名家庭经济困难大学生，为5000名大学生提供生源地助学贷款。支持铜川职业技术学院创新发展。深化医药卫生体制综合改革和城市公立医院综合改革试点，北京中医药大学第七临床医学院暨孙思邈医院投入运营，加快推进市人民医院二期工程建设，切实优化医疗服务。

　　加强公共文化建设。大力弘扬和践行社会主义核心价值观，加强“厚德陕西·德润铜川”宣传教育，在全社会营造崇德向善向上的浓厚氛围。国家公共文化服务体系示范区创建工作达到评估验收标准。加快推进铜川体育馆、铜川大剧院、铜川博物馆、工人文化宫、规划馆、科技馆等重大公共文化项目建设，建成全民健身馆，基层综合文化服务中心达标率达到80%。重视老年人体育工作，办好市第十一届运动会。

　　不断创新社会治理。深入推进平安铜川建设，按照“党政同责、一岗双责、齐抓共管、失职追责”的要求，夯实安全生产主体责任和监管责任，扎实开展安全专项整治和“打非治违”行动，加强安全文化建设，防范和遏制重特大事故发生。坚决落实“四个最严”要求，守护好人民群众“舌尖上的安全”。认真落实“十三五”突发事件应急体系建设规划，不断提升应急救援能力。加强信用体系建设，建立完善守信联合激励和失信联合惩戒机制，全面推行信用“红黑名单”制度，推动信用信息公开和共享，努力创建国家社会信用体系建设示范市。完善立体化社会治安防控体系，加大“严打”整治和大巡防工作力度，增强人民群众安全感。严格落实信访工作责任，依法做好矛盾纠纷排查化解工作。

　　各位代表，民之所望为施政所向。我们要把百姓摆在心中最高位置，把政府为民服务的责任尽足尽好，把各项民生实事办好办到位，让全市人民生活得更加幸福、更有尊严！

　　四、全面加强政府自身建设

　　打铁还需自身硬。面对艰巨繁重的任务，我们必须按照全面从严治党的要求，以更高标准、更严要求、更实作风，加快建设法治政府、创新政府、廉洁政府和服务型政府，不断提高政府治理能力现代化水平。

　　扎实推进依法行政。认真落实法治政府建设五年规划，把政府活动全部纳入法治轨道，全面依法履行政府职能。自觉接受市人大及其常委会的工作监督、法律监督和市政协的民主监督，认真听取各民主党派、工商联、无党派人士和人民团体的意见建议，高质量办好人大代表建议和政协提案。完善依法决策机制，坚持把公众参与、专家论证、风险评估、合法性审查、集体讨论决定作为重大行政决策法定程序，建立重大决策终身责任追究制度及责任倒查机制。深入实施“阳光政务”，全面推进决策、执行、管理、服务、结果“五公开”，方便群众知情、参与和监督。

　　扎实推进从严治政。加强政府系统党的建设，牢固树立“四个意识”，抓好机关作风建设，强化公务员教育管理，全面提升干部队伍素质。大力推行“互联网+政务服务”，推动网上政务大厅和实体政务大厅融合发展，让群众办事更加便捷。切实加强政府效能建设，弘扬“马上就办、真抓实干”作风，进一步压缩会议、精简文件、减少应酬，以踏石留印、抓铁有痕的执行力推动各项工作。认真落实“三项机制”，完善目标责任考核，强化督促检查、考核问责、正向激励，坚决向思想之尘、作风之弊、行为之垢开刀，激励广大干部把心思和精力集中在想干事、敢干事、会干事、干成事上。

　　扎实推进廉洁从政。认真执行“两准则四条例”，全面落实中央“八项规定”精神和国务院“约法三章”，进一步巩固整治“四风”成果。瞄准重点领域，加强廉政风险防控，进一步扎紧制度笼子。强化监督执纪问责，严厉查处各类违法违纪案件，坚决纠正损害群众利益的行为，无禁区、全覆盖、零容忍惩治腐败。加大公共资金、国有资产、国有资源、领导干部经济责任审计力度，做到应审尽审、凡审必严。厉行勤俭节约，严控“三公”经费，以政府过“紧日子”让群众和企业过“好日子”。

　　各位代表，上下同欲者胜，风雨共舟者兴。让我们更加紧密地团结在以习近平同志为核心的党中央周围，在省委、省政府和市委的坚强领导下，顽强拼搏，开拓进取，为加快 “四个铜川”建设、实现追赶超越目标而努力奋斗，以优异成绩迎接党的十九大和省第十三次党代会胜利召开！
